# Prevalence and dynamics of contraceptive use by type during the COVID-19 pandemic: Evidence from Western Iran

**DOI:** 10.1371/journal.pone.0300613

**Published:** 2024-03-19

**Authors:** Serajeddin Mahmoudiani

**Affiliations:** Department of Sociology and Social Planning, Shiraz University, Fars, Shiraz, Iran; Obafemi Awolowo University, NIGERIA

## Abstract

Contraception represents a deliberate choice made by individuals, both men and women, to regulate their desired number of children. The primary objective of this study was to examine the prevalence and predictors of contraceptive use, while also exploring the shifts in contraception methods following the COVID-19 pandemic. This study employed a quantitative approach with a survey technique. The survey was conducted in Kermanshah, one of Iran’s metropolises located in the western part of the country. The sampling methodology employed in this study involved a combination of multi-stage classification and systematic random methods. The survey took place between July and August 2022. The target population for the survey included women between the ages of 15 and 49. A total of 600 women from this population were selected and included in the survey sample. The sample was described using frequency tables, as well as central and dispersion indices (mean and standard deviation). Additionally, multivariate analysis was conducted through the application of logistic regression. Findings pointed out that approximately 65% of the women in the sample utilized contraception methods. Among these methods, the condom and oral pill were found to be the most prevalent choices. Moreover, the findings indicated that an increase in the number of both living and ideal children was associated with a decreased likelihood of contraceptive use. Following the occurrence of the COVID-19 pandemic, there was an observed increase in the utilization of traditional and natural methods of contraception. This shift highlights the importance of considering a broader range of contraceptive options and not solely focusing on restricting contraception services. In the midst of the coronavirus outbreak, women turned to traditional contraceptives, which may increase the risk of unintended pregnancies and subsequent miscarriages. Therefore, providing in-person services to women at their place of residence is necessary during epidemics.

## Introduction

Contraception is a crucial component of reproductive health and has long been a subject of significant attention from healthcare, demographic, and policy perspectives worldwide. It involves intentional measures undertaken by individuals, both male and female, to control and limit the number of children they have. Since the International Conference on Population and Development in 1994, reproductive health, including contraception, has been recognized as a fundamental human right [1, 2].

Contraception practice has a profound impact on various aspects of reproductive health. It influences birth intervals [3, 4], unintended pregnancies, and subsequently, induced abortions [5–8]. Additionally, contraceptive use plays a crucial role in determining the overall level of fertility [9–11] and has implications for maternal mortality. Furthermore, adopting contraception has been shown to reduce the risk of developing reproductive cancers, with oral contraceptive pills, for example, demonstrating a reduced risk of ovarian cancer [12, 13]. Conversely, limited access to contraceptives increases the vulnerability to feminine cancers [14].

Existing research has consistently highlighted various factors associated with contraceptive use, including educational attainment [15–19], age [18, 19], fertility preferences [18–21], monthly income [20], sex preferences [22, 23], mean age at marriage [24], employment status [24, 25], place of residence [26, 27], and access to family planning services [28]. These socioeconomic characteristics [29] collectively play a significant role in explaining the variations in contraceptive use. Similar findings [30–32] have also been observed in studies conducted within the context of Iran.

Following the release of the results of the 1966 census, Iran introduced its first-ever family planning program. The 1976 Fertility Survey of Iran revealed that approximately 36% of married women of reproductive age were utilizing contraception methods [33, 34]. However, after the 1979 Islamic Revolution, the family planning program was suspended by the Islamic Republic of Iran due to various factors such as the 1981 war with Iraq and increasing mortality rates. It was not until the 1986 census results were analyzed that the government initiated a second family planning program, with the aim of further reducing fertility levels in the country’s history.

Consequently, Iran witnessed a significant decline in fertility rates and a remarkable upsurge in contraceptive usage starting from 1989 onwards [35–42]. The 1989 report released by the Ministry of Health indicated that the contraceptive prevalence reached 49% during the period of 1976–1989. Furthermore, the prevalence of contraceptive use among married Iranian women aged 15–49 reached 64.5% in 1992 [33]. The findings from the 2010 Iran Demographic and Health Survey revealed that 77.4% of married Iranian women in their reproductive age utilized various forms of contraception [43].

The declining fertility rates and the attainment of below-replacement levels have prompted Iranian researchers and policymakers to reevaluate the future of population and family planning programs. In fact, in 2014, the supreme leader of Iran outlined population policies with a focus on promoting childbearing and achieving fertility levels higher than replacement levels. This present study aims to examine the prevalence of contraceptive use and enhance our understanding of the factors influencing its adoption or non-adoption. The study also seeks to provide insights for shaping Iran’s population policies. Additionally, this study aims to compare the usage of contraception methods before and after the COVID-19 pandemic, thereby shedding light on any changes or trends in contraceptive practices.

## Materials and methods

### Data and sample size

The data for this analysis was obtained from a cross-sectional survey conducted in Kermanshah, a city located in the western region of Iran. The utilized data is available in the supporting information section (S1 File). The survey was carried out between July and August 2022. Based on Iran’s most recent Population and Housing Census in 2016, the statistical population of interest comprised 283,298 women of reproductive age residing in Kermanshah city. Using a specific formula, the estimated sample size for this study was determined to be 600 women.

n = $\left[\frac{Nz^{2}pq}{Nd^{2}+z^{2}pq}\right]$ Z = 1.96, P = 0.5, d = 0.04, N = 283298

### Sampling

The sampling methodology employed in this study involved a combination of multi-stage classification and systematic random methods. Initially, the study population was divided into eight regions within Kermanshah. The sample size for each region was determined proportionately based on the population size of each respective region. As a result, eight statistical domains were selected to represent the entire area of Kermanshah, which consists of eleven districts. To facilitate the sampling process, maps were prepared for the selected areas within each region. Interviewers were trained on the sampling methods and were stationed within the designated blocks. They were instructed to position themselves in the northwest corner of a block and, while moving eastward, pass through the first two houses. Subsequently, they were to interview the residents of the third house if they met the eligibility criteria. Afterward, they would cross over to the previous two houses again and proceed to interview the occupants of the sixth house, continuing this pattern until the desired number of samples was achieved, effectively covering the entire block.

### Ethics considerations

The research project was confirmed by the Ethics Committee of Shiraz University of Medical Sciences, Shiraz, Iran, with the ethics code of IR.SUMS.REC.1399.1090. The participants were requested to provide a written informed consent. They were thoroughly briefed on the purpose and procedures of the study. Moreover, they were reassured regarding the strict confidentiality of their information.

### Measures

The data collection process involved the utilization of a structured questionnaire that was developed by the researchers. A total of 12 questions were included in the questionnaire, covering two main areas: demographic characteristics of the women (consisting of 7 questions) and various aspects related to fertility practices (comprising 5 questions).Both English and Persian versions of the questionnaire are available (S2 and S3 Files). In cases where the respondents were illiterate, our questioners assisted in completing the questionnaire on their behalf. To ensure the questionnaire’s validity, the content validity method was employed. Several experts specializing in reproductive health and demography fields assessed the questionnaire’s quality and confirmed its validity.

### Statistical analysis

For data analysis and result presentation, we utilized frequency tables and logistic regression analysis in the statistical software SPSS version 27. Frequency tables are employed for describing the sample. Central and dispersion indicators (mean and standard deviation) are utilized for quantitative variables, including age, age at first marriage, ideal and actual fertility. The analysis of the impact of each independent variable on contraceptive use probability, while controlling for other research variables, is conducted through bivariate logistic regression. To enable this analysis, qualitative independent variables like level of education and employment status are transformed into dummy variables and incorporated into the regression equation.

## Results

Table 1 presents an overview of the demographic characteristics of the respondents and their husbands. The average age of the women surveyed is approximately 36 years. Among the sample, 26.2% fall within the age range of 15–29, while 38.9% are aged between 30 and 39. Additionally, around 34.9% of the respondents are between the ages of 40 and 49. Furthermore, the average age at first marriage for the married women included in the study was approximately 20 years.

**Table 1:** Demographic and socioeconomic characteristics of the sample.

Variables	N(%)	Mean(SD)
Age (Year)		35.76(9.26)
15–29	157(26.2)	
30–39	234(38.9)	
40–49	209(34.9)	
Husband’s age (year)		41.94(10.06)
≤ 29	101(16.8)	
30–39	203(33.9)	
40–49	124(20.8)	
≥ 50	172(28.5)	
Women’s age at first marriage (Year)		20.44(4.33)
13–18	256(42.7)	
19–23	186(41.0)	
24–32	158(16.3)	
Educational level		
Illiterate	68(11.3)	
Primary	47(7.8)	
Secondary	54(9.0)	
High School	47(7.8)	
Diploma	154(25.7)	
University Degree	230(38.3)	
Husband’s educational level		
Illiterate	42(7.0)	
Primary	60(10.0)	
Secondary	35(5.8)	
High School	83(13.8)	
Diploma	85(14.2)	
University Degree	295(49.2)	
Employment status		
Employed	114(19.0)	
Housewife	486(81.0)	
Husband’s employment status		
Employed	491(81.8)	
Unemployed	109(18.2)	

The average age of the participants’ husbands is approximately 42 years, with a majority falling within the age range of 30–39. Among the surveyed women, around 11% are classified as illiterate, while about 38% have received higher education. Regarding employment status, during the time of the survey, approximately 19% of the women were employed, while the remaining participants were predominantly housewives. In contrast, around 82% of the respondents’ husbands were employed.

Table 2 provides insights into the ideal and actual fertility, as well as contraception practices, of the surveyed women. The most prevalent preference for childbearing among women is to have two children. On average, each woman desires to have 2.6 children. In contrast, the average number of children ever born, which serves as an indicator of actual fertility, is approximately 1.9 children per woman. According to the table, 54.7% of married women have between 1 and 2 children, while around 17% of them were childless at the time of the survey.

**Table 2:** Fertility and contraception practices of the sample.

Variables	N(%)	Mean(SD)
The number of ideal children		2.65(1.22)
0	7(1.2)	
1	58(9.7)	
2	273(45.5)	
3	126(21.0)	
4	95(15.8)	
≥ 5	41(6.8)	
The number of children ever born		1.97(1.57)
0	102(17.0)	
1	163(27.2)	
2	165(27.5)	
3	55(9.2)	
4	74(12.3)	
≥ 5	41(6.8)	
The use of contraceptive		
Yes	462(77.0)	
No	138(23.0)	
Contraception method among users before and after the COVID-19 incidence	Before	After
	N(%)	N(%)
Tubal Occlusion	52(11.3)	52(11.3)
Vasectomy	27(5.8)	27(5.8)
Oral Contraception Pill	156(33.8)	79(17.1)
Condom	100(21.6)	106(22.9)
Intrauterine Device (IUD)	27(5.8)	27(5.8)
Implant	18(3.9)	18(3.9)
Injection	34(7.4)	0(0.0)
Rhythm or Calendar Method	7(1.5)	21(4.5)
Withdrawal Method	28(6.1)	69(14.9)
Emergency Contraception Pill	13(2.8)	63(13.6)

The data reveals that over 77% of the women included in the study utilize contraceptives to restrict the number of children they have. Prior to the onset of the COVID-19 pandemic, the oral contraceptive pill and condom were the most frequently used methods of contraception among women. Following these two methods, tubal occlusion ranked as the third most popular choice among married women aged 15–49. Interestingly, the data also indicates that the Rhythm, emergency contraception pill and Implant methods had the lowest usage rates among women. However, after the COVID-19 incidence, the table highlights that the condom and oral contraceptive pill emerged as the first and second most common contraception methods respectively.

Following the COVID-19 pandemic, there has been a decline in the utilization of the injection method among women, decreasing from approximately 7% to around 0%. Conversely, the withdrawal, rhythm, and emergency contraception pill methods have witnessed an increase compared to the period prior to the COVID-19 incidence.

Table 3 displays the outcomes of the logistic regression analysis, with contraceptive use as the dependent variable. The results indicate that as the age of women increases, the odds of using contraceptives decrease. Also, the age of their husbands have a statistically significant negative effect on contraceptive use. The statistical analysis reveals that the age at first marriage for women has a statistically significant effect on the likelihood of using contraceptives. As the age of marriage increases, the likelihood of using contraceptives has decreased. There is a statistically significant relationship between actual fertility rate and contraceptive usage, indicating that as the number of children increases, the probability of using contraceptives also increases. Additionally, the findings suggest that as the number of ideal children a woman desires increases, the odds of using contraception decrease. The analysis reveals a statistically significant difference in the odds of contraceptive use among various education levels. Specifically, women with secondary education, and women with diploma education have lower odds of using contraceptives compared to women with university degree. The results of the logistic regression analysis indicate that women whose husbands have a secondary education are less likely to use contraception compared to women whose husbands have a university degree. Women whose husbands have a diploma are more likely to use contraception compared to women whose husbands have a university degree. The independent variables considered in the study have successfully predicted approximately 51% to 78% of the odds of contraceptive use among the participants.

**Table 3:** Prediction of contraceptive use through regression logistic.

Variables	B(Sig.)
Age	-0.347 (0.002)
Husband’s age	-0.203(0.007)
Age at first marriage	-0.248(0.018)
The number of children ever born	7.941(0.000)
The number of ideal children	-4.890(0.000)
Educational level	
Illiterate	9.246(0.999)
Primary	13.469(0.999)
Secondary	-6.105(0.000)
High school	10.055(0.998)
Diploma	-7.537(0.000)
University degree (Ref.)	
Husband’s educational level	
Illiterate	-28.655(0.998)
Primary	-4.089(1.000)
Secondary	-4.554(0.000)
High School	1.600(0.089)
Diploma	5.228(0.000)
University degree (Ref.)	
Employment status	
Housewife	-2.039(0.052)
Employed (Ref.)	
Husband’s employment status	
Unemployed	0.854(0.505)
Employed (Ref.)	

## Discussion

The decline in fertility rates in Iran since the mid-1980s, reaching below-replacement levels, has emerged as a significant concern for policymakers and authorities in Iranian society. This decline, from an average of around 7 children per woman to approximately 2 births per woman between 1985 and 2016, has sparked apprehension about the future of population growth and childbearing rates. In response to these concerns, the Government of Iran has expressed the intention to adopt and implement a pronatalist policy aimed at increasing fertility rates above replacement levels. The supreme leader of Iran has emphasized the importance of population policies focused on promoting higher childbirth rates. However, after six years, the reconsideration of population policies and their significance and necessity remain subjects of debate. The provision of family planning services, particularly free access to contraceptives, is a central point of controversy among demographers, health experts, and certain politicians. The continuity or discontinuity of such services has raised questions about the prevalence and predictors of contraception and their implications for new population policies. Therefore, the primary objective of this study was to investigate the prevalence and factors influencing contraceptive use, as well as explore the potential implications for the development of new population policies.

Consistent with previous research [44], the findings of the present study support the notion that the condom and oral contraceptive pill are the most commonly utilized methods of contraception. Moreover, an increase in women’s age was found to be associated with higher odds of contraceptive use, which is not consistent with the results of previous studies [18, 19, 30]. Additionally, an increase in the number of living children was associated with decreased odds of contraception, which is in line with previous research [18, 19, 31]. Similarly, the findings indicate that an increase in the number of ideal children was linked to a decrease in the probability of contraceptive use, as observed in earlier studies [18, 19, 30, 31]. Furthermore, consistent with previous research, the study revealed that fertility intentions play a role in the likelihood of contraceptive use. Lastly, having a university education was found to increase the likelihood of using contraceptive methods, consistent with prior studies.

The influence of educational level on the likelihood of contraceptive use has been observed in various contexts, confirming its consistent effect [15–19, 24, 45]. Moreover, our study revealed that approximately 17% of the target population utilized permanent contraception methods, including male and female sterilization. Additionally, around 10% of the participants employed long-acting reversible contraception methods, such as the implant and the IUD. Notably, the low prevalence of the rhythm method, also known as natural family planning, suggests that women in the study had limited knowledge about natural contraceptive methods. This finding highlights the need for increased awareness and education on natural contraception in the development of Iran’s new population policies. As mentioned earlier, this article primarily investigated changes in contraceptive use before and after the onset of the coronavirus pandemic. The results indicated that, in response to the COVID-19 crisis and the necessity to stay at home, women shifted towards traditional contraceptive methods. The utilization of methods like withdrawal and rhythm increased compared to the period before Corona. Conversely, the use of contraceptives such as pills, which required visiting relevant centers in person, decreased. The significant reduction in pill consumption suggested a shift. Decreasing the adoption of such methods and returning to traditional approaches, which carry a higher risk of failure, might have increased the likelihood of unintended pregnancy during the pandemic. Therefore, during epidemics, it is advisable to consider providing face-to-face services to women at their place of residence.

## Conclusions

It is crucial to acknowledge that reproductive health and access to contraception are fundamental human rights that should not be restricted. Women should have the freedom to choose and use any type of contraception whenever they desire. Empowering women through education and training can be effective in promoting the use of natural contraception methods (such as the Rhythm or Calendar method) that have fewer side effects.

In the Iranian community, the prevalence of masculine culture has resulted in a significantly lower utilization of male-related contraception methods, such as vasectomy, compared to female-related methods. To address this imbalance, it is important to encourage greater male involvement in reproductive planning within households. Including the participation of males in Iran’s new population policies is crucial. Iran’s demographic strategies underscore the aim of increasing fertility rates beyond the replacement level, with a specific focus on reaching 2.5 children per woman. The results of this study suggest that attaining this objective is feasible. Given that the ideal fertility aligns with the target outlined in Iran’s population policies, prioritizing the realization of women’s ideal fertility becomes crucial. Thoroughly examining the factors preventing women from reaching their ideal fertility demands careful consideration. By identifying and overcoming these challenges, there exists a significant likelihood of surpassing fertility rates beyond the replacement level. Hence, it is recommended that the current demographic policies in Iran prioritize achieving women’s ideal fertility rather than restricting access to contraceptive services.

According to the findings of this study, it can be asserted that, during epidemic outbreaks, a specialized program should be developed for women unable to visit health and treatment centers but in need of services. To achieve this, healthcare facilities can compile a list of women who sought family planning services before the pandemic, based on available records. These individuals did not seek services due to the need to stay at home during the outbreak. The lack of foresight for such epidemics may jeopardize maternal health, increasing the risk of unintended pregnancies and subsequent abortions. Therefore, it is recommended that relevant authorities incorporate the necessary planning and forecasts for epidemic times.

## Supporting information

S1 File Data. (SAV)

S2 File Questionnaire_English version. (PDF)

S3 File Questionnaire_Persian version. (PDF)
