# Supplementary material for: Prevalence and dynamics of contraceptive use by type during the COVID-19 pandemic: Evidence from Western Iran
Source: PLoS One. 2024 Mar 19;19(3):e0300613. doi: 10.1371/journal.pone.0300613 (PMC10950214; doi:10.1371/journal.pone.0300613)
Supplement: S2 File — (PDF) [file pone.0300613.s002.pdf]

Questionnaire (in English)

1. How old are you?...
2. How old is your husband?...
3. How old were you in your first marriage?...
4. Education level: Illiterate ☐, Primary ☐, Secondary☐, High school☐, diploma☐, University degree☐
5. Husband's education level: illiterate ☐ Primary ☐, Secondary☐, High school☐, diploma☐, University degree☐
6. Employment status: Employed☐, housewife☐
7. Husband's employment status: Employed☐, Unemployed☐
8. How many live children have you given birth to? ...
9. How many children do you think each couple should have?...
10. Do you use contraceptives? Yes☐, No☐
11. **Which contraceptives did you use before Corona?**

Female sterilization (Tubal Occlusion) ☐
Male sterilization (Vasectomy) ☐
Oral Contraception Pill☐
Condoms☐
Intrauterine Device (IUD) ☐
Implant☐
Injection☐
Breastfeeding☐
Rhythm or Calendar Method☐
Withdrawal Method☐
Emergency Contraception Pill☐

12. After corona, which method of contraception do you use?

Female sterilization (Tubal Occlusion) ☐
Male sterilization (Vasectomy) ☐
Oral Contraception Pill☐
Condoms☐
Intrauterine Device (IUD) ☐
Implant☐
Injection☐
Breastfeeding☐
Rhythm or Calendar Method☐
Withdrawal Method☐
Emergency Contraception Pill☐
