# Supplementary material for: Prevalence and dynamics of contraceptive use by type during the COVID-19 pandemic: Evidence from Western Iran
Source: PLoS One. 2024 Mar 19;19(3):e0300613. doi: 10.1371/journal.pone.0300613 (PMC10950214; doi:10.1371/journal.pone.0300613)
Supplement: S3 File — (PDF) [file pone.0300613.s003.pdf]

پرسشنامه (فارسی)

۱- چند سال دارید؟ ...

۲- همسر شما چند سال دارد؟ ...

۳- در اولین ازدواج چند ساله بودید؟ ...

۴- سطح تحصیلات: بی‌سواد☐، ابتدایی☐، راهنمایی☐، متوسطه☐، دیپلم☐، دانشگاهی☐

۵- سطح تحصیلات شوهر: بیسواد☐، ابتدایی☐، راهنمایی☐، متوسطه☐، دیپلم☐، دانشگاهی☐

۶- وضعیت اشتغال: شاغل☐، خانه دار☐

۷- وضعیت اشتغال شوهر: شاغل☐، بیکار☐

۸- در مجموع چند فرزنده زنده بدنیا اورده اید؟...

۹- بنظر شما امروزه هر زوج چند فرزند باید داشته باشد؟ ...

۱۰- آیا از وسایل پیشگیری از بارداری استفاده می کنید؟ بله☐، خیر☐

۱۱- قبل از کرونا از کدام وسایل پیشگیری از بارداری استفاده می کردید؟

عقیم سازی زنان (انسداد لوله ها)☐

عقیم سازی مردان (وازکتومی)☐

قرص ضد بارداری خوراکی☐

کاندوم☐

دستگاه داخل رحمی (IUD) ☐

ایمپلنت☐

تزریق☐

شیر دادن☐

روش ریتم یا تقویم☐

روش روش نزدیکی منقطع☐

قرص پیشگیری از بارداری اورژانسی☐

۱۲- بعد از کرونا از کدام سایل پیشگیری از بارداری استفاده می کنید؟

عقیم سازی زنان (انسداد لوله ها)☐

عقیم سازی مردان (وازکتومی)☐

قرص ضد بارداری خوراکی☐

کاندوم☐

دستگاه داخل رحمی (IUD) ☐

ایمپلنت☐

تزریق☐

شیر دادن☐

روش ریتم یا تقویم☐

روش نزدیکی منقطع☐

قرص پیشگیری از بارداری اورژانسی☐
